# Dual inhibition of HIV-1 replication by integrase-LEDGF allosteric inhibitors is predominant at the post-integration stage

**DOI:** 10.1186/1742-4690-10-144

**Published:** 2013-11-21

**Authors:** Erwann Le Rouzic, Damien Bonnard, Sophie Chasset, Jean-Michel Bruneau, Francis Chevreuil, Frédéric Le Strat, Juliette Nguyen, Roxane Beauvoir, Céline Amadori, Julie Brias, Sophie Vomscheid, Sylvia Eiler, Nicolas Lévy, Olivier Delelis, Eric Deprez, Ali Saïb, Alessia Zamborlini, Stéphane Emiliani, Marc Ruff, Benoit Ledoussal, François Moreau, Richard Benarous

**Affiliations:** 1 Biodim Mutabilis, Romainville 93230, France; 2 IGBMC, Inserm, CNRS, Université de Strasbourg, Illkirch 67404, France; 3 Institut Cochin, Inserm U1016, CNRS UMR 8104, Université Paris Descartes, Paris 75014, France; 4 LBPA, ENS Cachan, CNRS, Cachan, France; 5 CNRS UMR7212, Inserm U944, Université Paris Diderot, Conservatoire National des Arts et Métiers, Paris, France

**Keywords:** HIV, Integrase, LEDGF, Antiretroviral activity, Drug discovery, Allosteric inhibition, Protein-protein interaction inhibitor, Integrase inhibitor, Co-crystallization

## Abstract

**Background:**

LEDGF/p75 (LEDGF) is the main cellular cofactor of HIV-1 integrase (IN). It acts as a tethering factor for IN, and targets the integration of HIV in actively transcribed gene regions of chromatin. A recently developed class of IN allosteric inhibitors can inhibit the LEDGF-IN interaction.

**Results:**

We describe a new series of IN-LEDGF allosteric inhibitors, the most active of which is Mut101. We determined the crystal structure of Mut101 in complex with IN and showed that the compound binds to the LEDGF-binding pocket, promoting conformational changes of IN which explain at the atomic level the allosteric effect of the IN/LEDGF interaction inhibitor on IN functions. In vitro, Mut101 inhibited both IN-LEDGF interaction and IN strand transfer activity while enhancing IN-IN interaction. Time of addition experiments indicated that Mut101 behaved as an integration inhibitor. Mut101 was fully active on HIV-1 mutants resistant to INSTIs and other classes of anti-HIV drugs, indicative that this compound has a new mode of action. However, we found that Mut101 also displayed a more potent antiretroviral activity at a post-integration step. Infectivity of viral particles produced in presence of Mut101 was severely decreased. This latter effect also required the binding of the compound to the LEDGF-binding pocket.

**Conclusion:**

Mut101 has dual anti-HIV-1 activity, at integration and post-integration steps of the viral replication cycle, by binding to a unique target on IN (the LEDGF-binding pocket). The post-integration block of HIV-1 replication in virus-producer cells is the mechanism by which Mut101 is most active as an antiretroviral. To explain this difference between Mut101 antiretroviral activity at integration and post-integration stages, we propose the following model: LEDGF is a nuclear, chromatin-bound protein that is absent in the cytoplasm. Therefore, LEDGF can outcompete compound binding to IN in the nucleus of target cells lowering its antiretroviral activity at integration, but not in the cytoplasm where post-integration production of infectious viral particles takes place.

## Background

Raltegravir (Merck) and Elvitegravir (Gilead) were introduced in 2007 and 2012 respectively, as the first generation of integrase strand transfer inhibitors (INSTIs) and confirmed integrase (IN) as a clinically validated viral target for antiretroviral (ARV) therapy [1]. The mode of INSTI action was elucidated in complex with a retroviral IN for which the entire 3D structure was defined [2]. However, resistance to INSTIs has emerged in patients [3,4]. A second generation of INSTIs, less sensitive to drug-resistance mutations, has been approved (Dolutegravir (DTG) from GSK-Shionogi-ViiV). DTG belongs to the same class of compounds and remains sensitive to the strongest INSTI resistance mutations [5,6]. This highlights the need for integration inhibitors with completely different mechanism of action.

LEDGF/p75 (LEDGF), the main cellular cofactor of IN [7-9] is of great interest for the development of a novel generation of integration inhibitors. LEDGF interacts with IN through its C-terminal integrase binding domain (IBD). HIV-1 IN catalytic core (IN-CCD) and N-terminal domains are involved in the interaction with LEDGF [7-12]. LEDGF is crucial for integration and replication of HIV [13] although minor residual replication (~10%) was seen in LEDGF-depleted cells [14]. LEDGF functions as a tethering factor for IN, targeting the integration of HIV in actively transcribed gene regions of chromatin [15].

LEDGF binds to the interface of an IN dimer and promotes IN tetramerization which results in the functional form of IN required for concerted integration [16]. The elucidation of the 3D structure of the IN-LEDGF interfaces [11,12], together with the mapping of the critical residues involved [11,17] suggested the “druggability” of this target. The results defined a new IN pharmacophore which is different from the catalytic site targeted by existing INSTIs. A rational screening of the 3D structure by Zeger Debyser and colleagues resulted in the discovery of 2-(quinolin-3-yl) acetic acid derivatives (termed “LEDGINs”) as inhibitors of IN-LEDGF interactions [18]. Tert-butoxy-(4-phenyl-quinolin-3yl)-acetic acids (tBPQAs), analogues with closely related structures, have been identified by screening for inhibition of IN 3’ processing activity [19-22]. These tBPQAs are also efficient IN-LEDGF inhibitors. Several analogs to this family of molecules have since had patents submitted and published [23-34].

Several inhibitory activities of LEDGINs and tBPQAs have been reported so far. These include the inhibition of IN-LEDGF interaction, the inhibition of IN strand transfer and 3′ processing activities (independent of LEDGF), change in IN oligomerization toward stabilization of IN dimers and inhibition of the formation of the stable IN-viral DNA synaptic complex (SSC) [18,35-38]. These compounds are considered as allosteric inhibitors of IN that are able to block HIV integration [18,35-40] and are also referred to as ALLINIs [37,40]. These compounds remain fully active on IN mutants that are resistant to INSTIs and are therefore a promising new class of IN inhibitors. An inhibitory effect of LEDGINs on the infectivity of progeny virions has been reported lately [35,41-45]. The multiple activities of these compounds raise questions regarding the unicity or multiplicity of their mechanism of action. Here, we explore what mode of action could explain the multiple activities of these inhibitors. We investigate the respective contribution of these different activities to the overall ARV activity of these compounds using a new series of IN-LEDGF inhibitors from the LEDGIN and tBPQA family of compounds.

## Results

### Development of IN-LEDGF allosteric inhibitors

**Figure 1:**
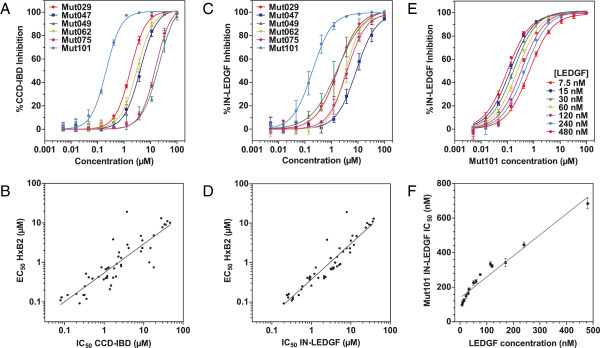
**Inhibition of IN-LEDGF interaction, correlation with ARV activity and LEDGF competition for IN binding. (A)** IN-CCD/LEDGF-IBD interaction inhibition dose–response curves of the compounds listed in Table 1. Data represent the means of six independent experiments with standard deviations shown as error bars. **(B)** Log-log correlation plot between IC_50_ of CCD-IBD interaction inhibition and EC_50_ of ARV activity for MT4 cells infected by HxB2 HIV-1. A subset of 51 Mut101 series compounds was studied (R^2^ = 0.77). **(C)** IN-LEDGF/p75 interaction inhibition dose–response curves. Data represent the means of three independent experiments with standard deviations shown as error bars. **(D)** Log-log correlation plot between IC_50_ of IN-LEDGF/p75 interaction inhibition and EC_50_ of ARV activity as in (B) (R^2^ = 0.88). **(E)** IN-LEDGF/p75 interaction inhibition dose–response curves for Mut101 at various LEDGF/p75 competing concentrations ranging from 7.5 nM to 480 nM. Data represent the means of two independent experiments done in quadruplicate with standard deviations shown as error bars. **(F)** Correlation plot between LEDGF/p75 concentration and IC_50_ of Mut101 IN-LEDGF interaction (R^2^ = 0.94).

**Figure 2:**
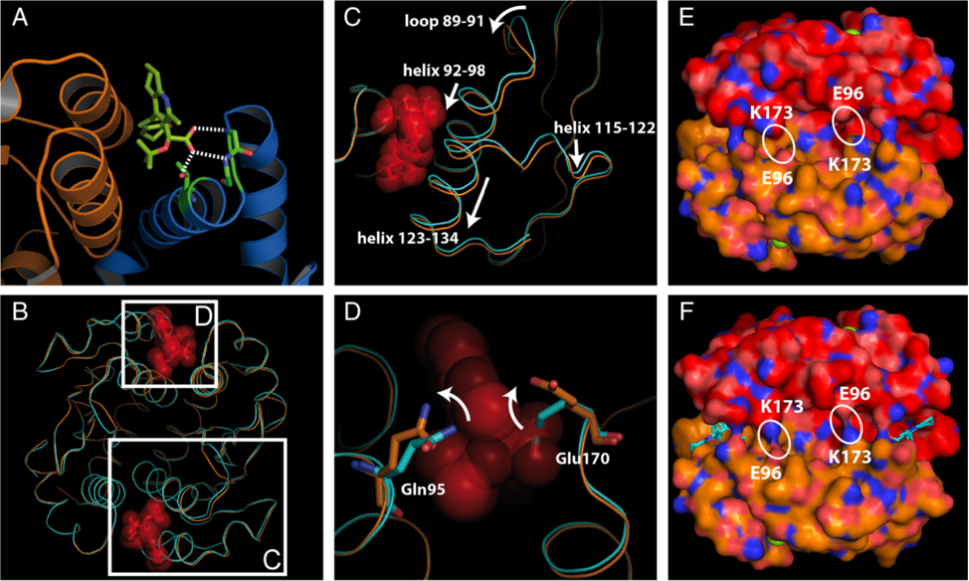
**Structure of Mut101 bound to IN-CCD. (A)** Zoomed view highlighting the hydrogen bonds between Mut101 (in green) and the IN-CCD dimer (in gold and blue). **(B)** Superimposition of the IN-CCD structures solved with (gold) and without (blue) Mut101. Two regions show significant differences and are highlighted by a white rectangle. **(C-D)** Enlargement of the two regions C and D showing the conformational changes upon ligand binding. **(E-F)** Solvent accessible surface coloring of IN monomers in red and gold, in absence **(E)** and in presence **(F)** of MUT101. Nitrogen atoms are in blue, oxygen in red and sulfur in yellow. Mut101 is represented in cyan. The position of K173 and E96 are shown on each monomer. The figure was made using PyMOL [73].

New IN-LEDGF allosteric inhibitors (INLAIs) of the aryl or heteroaryl-tertbutoxy-acetic acid family were designed. The structure and activities of 7 of these compounds are shown on Table 1. These compounds efficiently inhibited IN-CCD/LEDGF-IBD interaction as well as the interaction between IN and full length LEDGF proteins in homogeneous time-resolved fluorescence (HTRF) assays (Figure 1A and C). MT4 cells were infected with HxB2 HIV-1 and a subset of 51 compounds showed a good correlation between their ARV activity and their ability to inhibit IN-CCD/LEDGF-IBD or IN-LEDGF interactions (Figures 1B and D). The most active compound for IN-LEDGF inhibition, Mut101, also had the highest ARV activity (an EC_50_ value of 92 nM against HxB2 infection, CC_50_ for cytotoxicity was undetectable at over 50 μM (Table 1)). LEDGF was able to compete with these inhibitors, increasing the IC_50_ of Mut101 on IN-LEDGF interaction inhibition from 0.097 to 0.68 μM (Figure 1E-F). Mut101 and several of these inhibitors were co-crystallized with the IN-CCD dimer, showing that their binding pocket on IN corresponds to the LEDGF-binding site (Figure 2A). Data collection and refinement statistics are given on Additional file 1: Table S1. Two Mut101 molecules bound to the IN-CCD dimer (Additional file 1: Figure S1A). The ligand was found to be in a pocket surrounded by hydrophobic residues on one side, an acidic region on the other side and basic residues at the bottom of the pocket (Additional file 1: Figure S1B). Three hydrogen bonds link the carboxylic acid group of Mut101 and the protein (Figure 2A), one with the hydroxyl group of the side chain of Thr 174, and two with the amino group of the main chain of His 171 and Glu 170. In addition Mut101 was found to interact with two water molecules (Additional file 1: Figure S1C). The IN-CCD structures with and without Mut101 were superimposed. We found structural differences that appear in two regions (Figure 2B), which contrasts with previously reported IN-CCD/LEDGIN or tBPQA co-structures where no differences were found [18,36,37]. The first region of structure difference encompasses alpha helices 115–122 and 123–134 as well as the alpha helix 92–98. Surprisingly, a strong displacement of the loop encompassing residues Ile 89, Pro 90 and Ala 91 was found to affect the two monomers (Figures 2C). The same differences have been observed with the IN-CCD/LEDGF-IBD structure [11]. The second region of difference is in the Mut101 binding pocket where the side chains of Gln 95 and Glu 170 are displaced (Figure 2D). These long range structural changes are affecting the IN catalytic site, see movie in supplementary (Additional file 2, the catalytic site is at the position of the magnesium ion shown as a green sphere), which explains the decrease in the 3′ processing activity in the Mut101 bound form of IN. Upon ligand binding, conformational changes in the dimerization interface lead to stronger interactions, stabilizing the IN dimer. For example, the side chains of Gln 96 and Lys 173 are interacting in the presence of Mut101 as shown in Figure 2E-F and in the supplementary movie (Additional file 2). These interactions strongly stabilize the IN dimeric form and explain the multimerization effect with the binding of Mut101. Moreover, the structural changes at the IN surface upon Mut101 binding most probably affect IN interaction with protein cofactors and DNA. Altogether these results confirm and explain at the atomic level the allosteric effect of the IN/LEDGF interaction inhibitor.

**Table 1:** Structure and activity of IN-LEDGF inhibitors designed in this study

**Compound**	**Structure**	**MW (g/mol)**	**Biochemical assays**			**MT4 assays**		
			**CCD-IBD IC**_**50 **_**(μM)**	**IN-LEDGF IC**_**50 **_**(μM)**	**IN ST IC**_**50 **_**(μM) plateau (%)**	**NL4-3 EC**_**50 **_**(μM)**	**HxB2 EC**_**50 **_**(μM)**	**CC**_**50 **_**(μM)**
Mut029*		391	1.7	2.5	NT	3.8	3.8	>50
Mut047*		390	3.8	7.9	0.88 70%	16	19	>50
Mut049		355	18	3.5	0.18 56%	2.0	0.75	>50
Mut062*		394	2.7	3.1	0.54 73%	3.3	1.3	>50
Mut063		433	>100	>100	>100 ND	>50	NT	>50
Mut075		353	14	4.0	NT	3.4	1.0	>50
Mut101*		410	0.23	0.20	0.17 66%	0.54	0.092	>50

Structure, molecular weight, and activities of compounds. NT = not tested. *compounds that have been co-crystallized with IN-CCD; IC_50_ = concentration required to inhibit CCD-IBD interaction, IN-LEDGF interaction or IN strand transfer activity by 50%; EC_50_ = concentration required to inhibit HIV-1 infection of MT4 cells by 50%; CC_50_ = concentration required to inhibit MT4 cell viability by 50%.

### Effect of IN-LEDGF inhibitors on IN strand transfer and 3’ processing activities is independent of LEDGF

**Figure 3:**
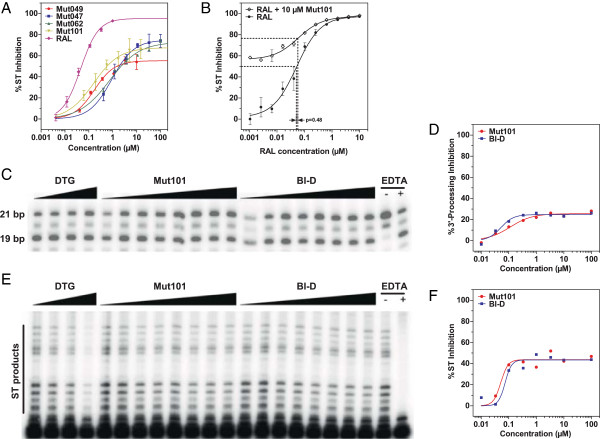
**Effect of INLAIs on IN catalytic activities. (A-B) IN strand transfer inhibition in ELISA assay: (A)** The IN strand transfer inhibition of compounds listed in Table 1 is compared to inhibition with Raltegravir (RAL). Data represent the means of three independent experiments with standard deviations shown as error bars. **(B)** Additive effect of Mut101 and Raltegravir on IN strand transfer inhibition. Comparison of dose–response curves of Raltegravir alone and Raltegravir in the presence of 10 μM Mut101. Mean of triplicate with standard deviation. Dotted lines highlight the IC_50_ of Raltegravir in both conditions (difference not significant, Student’s t-test p = 0.48). **(C-D) IN 3′ processing inhibition by Mut101 and BI-D assayed using standard radioactive assay:** increasing concentration of Dolutegravir (DTG, from 3.3 to 100 nM), BI-D or Mut101 (from 0.01 to 100 μM) were used. The relative cleavage efficiency is reported for BI-D and Mut101 **(D)**, and corresponds to the ratio between the product (19 bp) and the substrate (21 bp) converted to % inhibition. DTG resulted in 16% inhibition at 100 nM. **(E-F) IN Strand transfer inhibition activity of Mut101 and BI-D assayed using standard radioactive assay:** increasing concentration of DTG (from 0.3 to 10 nM), BI-D or Mut101 (from 0.01 to 100 μM) were used. The relative strand transfer efficiency is reported for BI-D and Mut101 **(F)**, and corresponds to the ratio between the strand transfer products depicted on the autoradiography and the substrate (19 bp), converted to % inhibition. DTG has an IC_50_ of 2.7 nM.

We found that these compounds inhibited the IN strand transfer activity as quantitated by ELISA assay (Figure 3A), in agreement with previously reported data, with IC_50_ values in a similar range to those found for inhibition of the IN-LEDGF interaction (Table 1). Activity in the concentration range studied (up to 100 μM) was always partial (reaching a plateau at 56-73% inhibition), which contrasts the full inhibition obtained using Raltegravir. In contrast with data reported by Christ *et al. *[35], modification of the order of addition of compounds, before or after DNA in this strand transfer assay, did not result in full inhibition (data not shown). This partial and weaker inhibition than that of INSTIs, was confirmed using a typical assay with radioactive oligonucleotide and gel analysis of the strand transfer products (Figure 3E-F). Mut101 and Raltegravir had an additive inhibitory effect on IN strand transfer activity: there was no significant change in the IC_50_ value of Raltegravir in the presence of a saturating concentration of Mut101 (52 nM *vs.* 58 nM when both present; Student′s t-test: p = 0.48; Figure 3B). This IN strand transfer inhibition was found regardless of whether or not the donor DNA was preprocessed [36]. Inhibition of IN 3′ processing activity was reported for some INLAIs [37]. We found that increasing concentrations of Mut101 or BI-D lead to a slight decrease in the 3′ processing efficiency (with a maximum of 25-30% inhibition, Figure 3C-D), but their inhibition of the IN strand transfer reaction was more important. (Figure 3E-F).

### IN-LEDGF inhibitors enhance the IN-IN interaction

**Figure 4:**
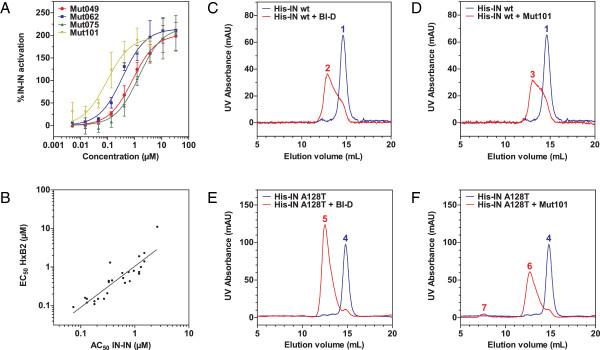
**Effect of INLAIs on the oligomeric state of IN. (A-B) IN-IN HTRF interaction: (A)** IN-IN interaction activation dose–response curves. Data represent the means of three independent experiments done in duplicate with standard deviations shown as error bars. **(B)** Correlation between AC_50_ of IN-IN interaction and EC_50_ of ARV activity on MT4 cells infected with HxB2 HIV-1 (R^2^ = 0.78). This study used 21 of the set of 51 compounds. AC_50_ = concentration required to activate IN-IN interaction by 50% of the maximum effect. **(C-F) Size exclusion chromatography of IN:** Binding of INLAIs BI-D or Mut101, to IN NL4-3 wt **(C-D)** or IN NL4-3 A128T **(E-F)**, promotes a shift toward higher IN oligomeric state, independently of LEDGF. Blue peaks: elution of IN wt and IN A128T in the absence of compound. Red peaks: elution of IN wt and IN A128T in the presence of BI-D **(C, E)** or Mut101 **(D, F)**.The elution volume and identification of each peak (numbered 1 to 7) are indicated in supplementary table S2.

We evaluated the ability of IN-LEDGF inhibitors to promote modifications in the interaction between IN subunits as these inhibitors act at the IN dimer interface. We designed an HTRF-based assay to monitor the interaction between His_6_-IN/Flag-IN subunits. In the presence of compound concentrations the HTRF signal corresponding to the His_6_-IN/Flag-IN interaction was more than twice as strong as the signal obtained in the absence of compound (Figure 4A). The concentration required to activate the IN-IN interaction by 50% (AC_50_) closely correlated with the inhibition of the IN-LEDGF interaction and the antiretroviral activity EC_50_ (Figure 4B). Raltegravir had no effect on either the IN-LEDGF interaction or IN-IN interaction (data not shown). These results are in agreement with previously reported observations on the effect of some LEDGINs and tBPQAs on IN-IN interactions [35-37]. In order to determine if this enhancement of IN-IN interaction corresponds to a change toward higher IN oligomerization state, we performed size exclusion chromatography of IN that has been or not preincubated with Mut101 or with the related compound BI-D. As shown in Figure 4C-D and on Additional file 1: Table S2 for the elution volumes of the different peaks, while IN wt in the absence of INLAIs behaves as an IN dimer (blue peaks), pre-incubation with Mut101 or BI-D results in higher IN oligomerization state (red peaks), that likely corresponds to a partial formation of IN tetramer. Raltegravir had no effect (data not shown). In contrast with some LEDGINs previously described [18], Mut101 and BI-D conserved full ARV activity on the HIV-1 mutant IN A128T and full in vitro activity on the IN NL4-3 A128T protein mutant. So, we performed similar experiments with this IN A128T protein. As shown in Figure 4E-F and on Additional file 1: Table S2, the higher IN oligomerization state promoted by binding of Mut101 or BI-D to the LEDGF binding pocket, corresponds clearly to a shift from IN dimer (blue peaks) toward IN tetramer (red peaks). This slight difference between the results obtained with IN wt and the IN A128T mutant is likely due to a more soluble behavior of the IN A128T mutant protein compared to IN wt. In both experiments we did not observe the formation of IN aggregates of very high molecular weight, except for a very minor peak (peak 7) after incubation of IN A128T with Mut101, which elution volume (see Additional file 1: Table S2) could correspond to the formation of such aggregates. However, we cannot exclude that insoluble aggregates are formed but do not enter the gel filtration matrix.

Altogether, wee confirmed that, in addition to their ability to inhibit IN-LEDGF, IN-LEDGF inhibitors are allosteric inhibitors of IN and promote IN conformational change by binding to the LEDGF-binding pocket and mimicking the effect of LEDGF binding to IN [16,46].

### Mut101 behaves as an inhibitor of integration in time-of-addition experiments

**Figure 5:**
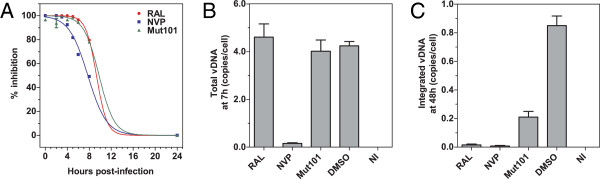
**Time of addition experiment and qPCR analysis. (A)** Time-of-addition (TOA) experiment in single-round infection assay of MT4 cells infected with VSV-G-pseudotyped HIV-1 Δenv-Luc NL4-3. Each compound (Mut101, Nevirapine (NVP) or Raltegravir (RAL)) was added at the indicated times post-infection. Infection was measured by luciferase assays. Relative inhibition (%) was determined by comparison with the control. qPCR analysis of **(B)** the formation of proviral DNA at reverse transcription, and **(C)** the integration of the proviral DNA in the host cell genome after compound treatment as indicated. NI: not infected. Data represent the means of quadruplicates with standard deviations shown as error bars.

We performed a time-of-addition experiment (TOA) to identify the HIV-1 replication cycle step that is blocked by Mut101. We used Mut101 at a saturating concentration (25 μM) and single-cycle infection kinetics with VSV-G-pseudotyped Δenv HIV-1 NL4-3 expressing luciferase as a measure of infection. The kinetics of decreased activity after Mut101 addition were very similar to that observed with Raltegravir, but different to those of Nevirapine, suggesting that Mut101 at saturating concentration behaved as an inhibitor of integration (Figure 5A). This is in full agreement with data reported previously on LEDGINs and tBPQAs [18,36]. The replication cycle analysis by quantitative PCR confirmed that Mut101 inhibited the integration of the proviral DNA (Figure 5C) but not the production of proviral DNA at reverse transcription (Figure 5B).

### Mut101 remains fully active against HIV-1 mutants that are resistant to INSTIs and other anti-HIV drugs

Mut101 was tested against a panel of virus mutants harboring, in an NL4-3 background, some of the strongest resistant mutations to INSTIs and other classes of ARV drugs used in clinics [47]. These mutants are listed on Table 2. The activity of Mut101 and reference compounds was quantified by the fold change (FC) ratio between EC_50_ on resistant virus and EC_50_ with the wild type (wt) – a measure of compound efficacy on resistant mutant virus. Mut101 had an FC ratio of 1 or lower against all resistant viruses contrasting the results with reference compounds (Table 3). This demonstrates that Mut101, as IN-LEDGF inhibitor, is a candidate for a novel class of drugs that can act on viruses resistant to those currently used in clinics, including INSTIs.

**Table 2:** Resistant viruses used in this study

**Resistance mutations to**	**Gene**	**Mutations**
Protease inhibitor (PI)	Protease	L10R, M46I, L63P, V82T, I84V
Nucleoside RT inhibitor (NRTI)	RT	M41L, D67N, T69N, K70R, T215F, K219E
Non-nucleoside RT inhibitor (NNRTI)	RT	K103N, Y181C
Nucleoside and non-nucleoside RT inhibitor (Multi-drug)	RT	M41L, D67N, K103N, M184V, L210W, T215Y
Integrase strand transfer inhibitor (INSTI)	Integrase	G140S, Q148H

Mutant viruses used in this study that are resistant to PIs, NRTIs, Multi-drugs (NRTI + NNRTI), NNRTIs or INSTIs.

**Table 3:** EC50 fold-changes on resistant viruses

**Compound**	**EC**_**50 **_**(μM)**	**EC**_**50 **_**fold change**				
	**NL4-3 WT**	**PI**	**NRTIs**	Multi-drug*	**NNRTIs**	**INSTIs**
RAL	0.007	1	1	0.5	1	374
EVG	0.003	1	0.3	0.3	1	2036
AZT	0.14	1	88	10	0.1	0.4
NVP	0.16	1	3	14	287	1
IDV	0.032	9	1	1	1	1
*Mut101*	0.47	*1*	*1*	*1*	*1*	*1*

EC_50_ fold change (FC) of Mut101, Raltegravir (RAL), Elvitegravir (EVG), Nevirapine (NVP), Zidovudine (AZT) and Indinavir (IDV) (ratio of the EC_50_ on resistant viruses to the EC_50_ on the wt virus). Data represent the mean of two independent experiments. * contains resistance mutations against NRTIs and NNRTIs.

### Unlike INSTIs, the Mut101 series of compounds are more potent when assayed with replicative HIV-1 than with non-replicative pseudotyped virus

The ARV activity of a drug can be assessed using different assays. Multiple round infection using a replication-competent virus reveals the global ARV activity of a drug, but cannot give an indication as to which step of the viral replication cycle is blocked. All classes of drugs are found fully active in multiple round infection assays. In contrast, in single-round infection, replication-defective Δenv viruses pseudotyped with an exogenous envelope (VSV-G) can complete viral replication only up to integration. This enables drugs like RT or IN inhibitors (fully active because they act early during the replication cycle, before or at integration) to be distinguished from drugs such as protease inhibitors that act late after integration (inactive in the single cycle assay) (see Table 4).

**Table 4:** Antiviral activities in single-round and multiple-round infection assays

**Drug**	**NL4-3 EC**_**50 **_**(μM)**		**SR/MR ratio**
	**Single-round**	**Multiple-round**	
EFV	0.0012 ± 0.0004	0.0013 ± 0.0002	0.9
RAL	0.0025 ± 0.0003	0.0024 ± 0.0006	1
EVG	0.00042 ± 0.00005	0.00086 ± 0.0004	0.5
NVP	0.041 ± 0.008	0.086 ± 0.012	0.5
AZT	0.0025 ± 0.0003	0.0024 ± 0.0006	0.6
IDV	Inactive	0.036	NA
SQV	Inactive	0.013	NA
Mut029	30 ± 4	3.8 ± 1.6	8
Mut047	37 ± 2	16 ± 7	2.3
Mut049	41 ± 2	2.0 ± 0.1	21
Mut062	30% @50	3.3 ± 1.5	>25
Mut075	23% @50	3.4 ± 1.0	>25
Mut101	9.0 ± 1.5	0.49 ± 0.04	18

EC_50_ for the ARV activity of Mut101 compound series (tested in SR and MR assays) and the SR/MR ratio compared to the indicated drugs. Data represent the mean of six independent experiments.

**Figure 6:**
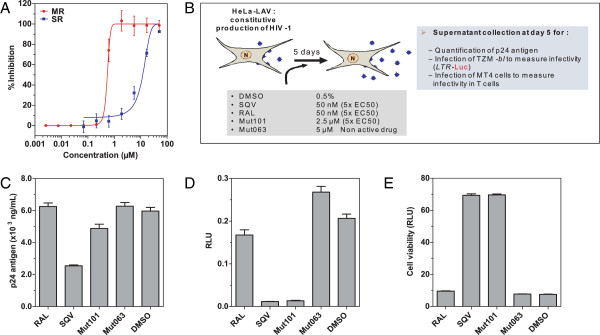
**ARV activity in infection assays and effect on the infectivity of virions produced by HeLa-LAV cells. (A)** Plot comparing the ARV activity of Mut 101 tested by MR and SR infection assays (using the same concentration scale). **(B)** Diagram of the experimental setup used to study infectivity of virions produced by HeLa-LAV cells that were treated with Mut101, Raltegravir, SQV, Mut063 or DMSO at the indicated concentrations. **(C)** Titration of p24 harvested from HeLa-LAV cells treated with the indicated compounds. **(D)** Infectivity of virions harvested from HeLa-LAV cells treated with the indicated compounds and tested by infection of TZM indicator cells and luciferase assay. **(E)** Infectivity of virions harvested from Hela-LAV cells treated with the indicated compounds and tested by infection of MT4 cells and cytopathic assay using CellTiter-Glo®.

Drugs that act early during reverse transcription (such as AZT and Nevirapine), or at integration (such as Raltegravir) showed ARV activity that is similar or slightly better in single-round (SR) infection assays than in multiple round (MR) infection assays (an EC_50_ SR/EC_50_ MR ratio of 1 or lower; Table 4). IN-LEDGF inhibitors, as allosteric inhibitors of HIV-1 integrase, were expected to behave similarly to Raltegravir with a SR/MR ratio close to 1. Intriguingly this was not the case. In contrast, Mut101 and the other compounds of this study were much more potent in MR than in SR infection assay with EC_50_ SR/EC_50_ MR ratios always much higher than 1 and up to 18 for Mut101 (Table 4). Mut101 and the other IN-LEDGF inhibitors also differ from protease inhibitors (PIs) since PIs are active only in MR and completely inactive in SR assays. The Mut101 series of IN-LEDGF inhibitors have an unprecedented mixed profile with moderate ARV activity in SR and more potent activity in MR infection assays. The two dose–response curves of Mut101 ARV showed that there was no or minimal activity detectable in the SR assay at the concentration resulting in maximum MR activity (Figure 6A). This suggests that the contribution of integration inhibition (estimated by SR assay) to Mut101 overall ARV activity is minimal at this concentration. This contribution becomes significant only at much higher concentrations, such as those used for TOA experiments. Previous infection experiments studying LEDGINs and tBPQAs ARV activity were performed mostly in MR assay. We analyzed the behavior of a tBPQA, racemic BI-D [48] (structure shown in Additional file 1: Figure S2), to determine if the behavior of the Mut101 compound series is shared by other LEDGINs and tBPQAs. We found a similar discrepancy between high EC_50_ in SR (2.4 μM) and much lower EC_50_ (0.17 μM) in MR assay.

### Mut101 also promotes a post-integration block producing defective HIV-1 progeny virions

The discrepancy between potent ARV activity in MR assays and moderate activity in SR assays, distinguishes Mut101 from INSTIs that specifically block HIV integration. One explanation could be that Mut101 treatment results in a second ARV activity at a late stage of the replication cycle, post-integration. We used the HeLa-LAV system in which the HeLa cell line has been transduced by HIV-1 LAV virus [49] to test this hypothesis. HIV-1 LAV is constitutively integrated in this cell line and HeLa-LAV cells produce HIV-1 LAV virions that cannot reinfect the cells as they do not express CD4 on their surface. Only drugs that could block virus production at the post-integration step of the HIV-1 replication cycle are expected to be active in this cell line. We treated HeLa-LAV cells with Mut101, Raltegravir, Saquinavir (SQV) or DMSO (as a negative control). The infectivity of viruses produced in the presence of these compounds was tested in TZM indicator cells expressing luciferase and by infection of MT4 cells. The design of this experiment is schematized in Figure 6B. The amount of p24 produced with virus treated by Mut101 was comparable to viruses treated with Raltegravir, DMSO or Mut063 an inactive analogue of Mut101 (Figure 6C). In contrast, luciferase assay in TZM cells showed that Mut101 and SQV treatments resulted in strong virus infectivity defects; viruses produced in the presence of Raltegravir, DMSO or Mut063 had no infectivity defect (Figure 6D). These results were confirmed by determining the cytopathic effect of infected MT4 cells using a CellTiter-Glo® assay (Figure 6E). The infectivity defect was not due to a residual concentration of Mut101 used during virus production since the virus stock was diluted 2000 times, to an inefficient concentration much below its EC_50_. We can also rule out a virucidal effect of Mut101 on virus particles released in the supernatant as Mut101 was unable to inactivate free virus once released in the supernatant of producing cells. Altogether, these results are strongly in favor of a defect provoked at a post-integration step by Mut101 treatment. This defect is additional to the block at integration detected above by the TOA experiment. Western blot using anti-p24 antibody did not detect any perturbation of Gag maturation and CA p24 content in defective virions or in Mut101-treated HeLa-LAV cell lysates (data not shown).

### A post-integration defect promoted by Mut101 treatment requires Mut101 binding to the LEDGF-binding pocket of IN

**Figure 7:**
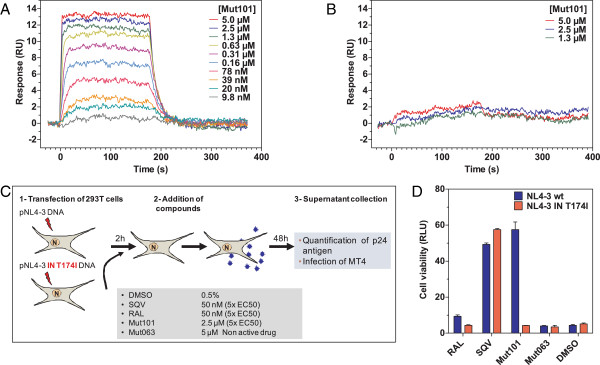
**Binding to IN-CCD wt, T174I mutant, treatment during production of wt and T174I mutant viruses. (A)** Binding kinetics of Mut101 to IN-CCD wt. Serial dilutions of Mut101 (between 9.8 nM and 5 μM) were injected on immobilized GST-Flag-CCD wt. **(B)** Binding kinetics of Mut101 to IN-CCD T174I. Serial dilutions of Mut101 (between 1.3 μM and 5.0 μM) were injected on immobilized GST-Flag-CCD T174I. **(C)** Diagram of the experimental setup to study the infectivity of NL4-3 wt and IN T174I virions produced after transfection of 293 T cells. The indicated compounds were added during virus production for 48 h. Supernatants were tested for virus production by p24 assay, and for virus infectivity. **(D)** Infectivity of wt NL4-3 (blue bars) and IN T174I NL4-3 mutant (red bars) virions harvested from 293 T transfected cells after treatment with the indicated compounds and infection of MT4 cells by cytopathic assay using CellTiter-Glo®.

The post-integration block promoted by Mut101 cannot be explained by impaired IN-LEDGF interaction or the inhibition of IN catalytic activity. It could be suggested that such a post-integration defect might be related to an unknown Mut101 target, in addition to IN. We generated an NL4-3 HIV-1 virus bearing the T174I mutation in the LEDGF-binding pocket of IN to rule out this hypothesis. We (E. Le Rouzic unpublished results) and others [36] have selected the T174I mutation for resistance to IN-LEDGF inhibitors: Mut101 had an EC_50_ > 50 μM on this mutant compared to an EC_50_ = 0.49 μM on NL4-3 wt. We used Surface Plasmon Resonance (SPR) to confirm that Mut101 was less able to bind to the mutated IN-CCD T174I than to IN-CCD wt. Mut101 bound to IN-CCD wt with high affinity (K_d_ = 0.12 μM) in a similar range to the IC_50_ or AC_50_ found in HTRF assays for inhibition of the IN-LEDGF interaction or enhancement of the IN-IN interaction, respectively (Figure 7A). Mut101 had no significant binding to the mutated IN-CCD T174I (Figure 7B). HIV-1 NL4-3 wt and the NL4-3 IN T174I mutant virus were produced by HEK293T cell transfection in the presence of Mut101, SQV, Raltegravir, Mut063 or DMSO. Virions were harvested and used to infect MT4 cells (as schematized in Figure 7C); their infectivity was tested using a cytopathic CellTiter-Glo® assay. As shown in Figure 7D, NL4-3 wt virus (blue bars) produced in the presence of Mut101 was inactivated and the viability of MT4 cells infected by this virus was preserved. In contrast, the mutant virus T174I (red bars) was insensitive to Mut101 treatment and MT4 cells were fully infected and their viability abrogated. Both wt and T174I viruses were sensitive to and inactivated by SQV treatment. Raltegravir treatment during virus production had no effect on either virus; these retained full infectivity which was comparable to that observed after DMSO or Mut063 treatment. These results demonstrate that integrase is indeed the unique target of Mut101 for its ARV activity, both at the integration and post-integration steps of the HIV-1 replication cycle.

## Discussion

IN-LEDGF allosteric inhibitors (INLAIs) are a new class of IN inhibitors whose binding site, the LEDGF-binding pocket, is different from the IN catalytic site targeted by INSTIs. In this study we described new IN-LEDGF inhibitors from the family of LEDGINs and TBPQAs. These compounds shared multiple activities with the previously described compounds of this class. These include: inhibition of the IN-LEDGF interaction, weak inhibition of IN strand transfer activity (additive to that of Raltegravir) and even weaker inhibition of IN 3′ processing activity, IN conformational change by increased IN-IN interaction that favors higher order oligomerization state of IN (independent of LEDGF, with AC_50_ similar to the IC_50_ found for IN-LEDGF inhibition), and a dual mode of ARV activity at both integration and post-integration steps of viral replication. These results define the Mut101 series of compounds like other IN-LEDGF inhibitors as *bona fide* allosteric inhibitors of IN functions. Since both catalytic activities of IN, 3′ processing and strand transfer are dependent on the oligomeric state of IN [50], it is likely that the shift of IN dimer toward higher order oligomeric state of IN promoted by Mut101 or BI-D binding, is more detrimental to the strand transfer reaction rather than to the 3′ processing activity of IN.

Our co-crystallographic studies with Mut101 bound to IN-CCD allowed us to detect conformational changes resulting from compound binding in the binding site of inhibitors. The structural changes observed when Mut101 is bound to IN confirm and explain the allosteric effect of the IN/LEDGF interaction inhibitor which acts at the post-integration steps. We evidenced a direct correlation between allosteric changes with atomic details and functional effect on IN upon Mut101 binding. Our experiments enabled us to address important questions regarding the unicity or multiplicity of the mechanism of action of these inhibitors, the respective contributions of these inhibitory activities to overall ARV activity, and the specific mode of action of these new ARV agents.

Blocking at integration can be explained by the inhibition of IN strand transfer and IN-LEDGF interaction, given the role of LEDGF in the tethering of IN to chromatin during the integration process of HIV-1. The post-integration block promoted by INLAIs is not in line with these activities. This raises the possibility that these compounds have another unrelated target in addition to the LEDGF-binding pocket of IN. We ruled out this hypothesis using a virus mutated in the LEDGF-binding pocket of IN (NL4-3 IN T174I, resistant to Mut101 ARV activity) and demonstrate that IN is indeed the target of this post-integration defect: the lack of Mut101 binding to the IN-CCD T174I correlated with the absence of effect of Mut101 on the production of the NL4-3 IN T174I mutated virus. We conclude that both the integration and post-integration blocks promoted by INLAIs are related to the binding of these compounds to a unique target, the LEDGF-binding pocket of IN. This dual inhibitory activity, at two different steps of the HIV-1 replication cycle through the same viral target, is unprecedented for all classes of ARV drugs.

We investigated the respective contributions of the two mechanisms to the global ARV activity of these compounds. SR infection assays reflect the activity of an ARV compound during an early step of the HIV replication cycle (up to integration), and MR infection assays reflect global ARV activity. We showed that the post-integration inhibition of the HIV-1 replication cycle is the major mechanism contributing to global Mut101 ARV activity. There was no or minimal ARV activity detectable in SR infection assay at the same Mut101 concentration that achieved 100% inhibition of HIV-1 infection in the MR infection assay. A higher concentration of Mut101 was required to detect ARV activity in the SR assay since its EC_50_ in this format (9 μM) was 18 times higher than its EC_50_ in MR infection assay (0.49 μM). TOA experiments used a Mut101 concentration (25 μM) that was high enough to permit 100% of ARV activity in the SR infection assay. Our study demonstrates that Mut101 and the other INLAIs of this series are not acting mainly as inhibitors of HIV-1 integration. This is in contrast to early studies reported on LEDGINs, based on MR infection experiments performed at saturating inhibitor concentration, that suggested they act as integration inhibitors [18]. HIV-1 integrase is the unique target of Mut101 for its ARV activity. However, the major action of Mut101 and other related INLAIs is as post-integration inhibitors producing defective infectious HIV-1 virions.

**Figure 8:**
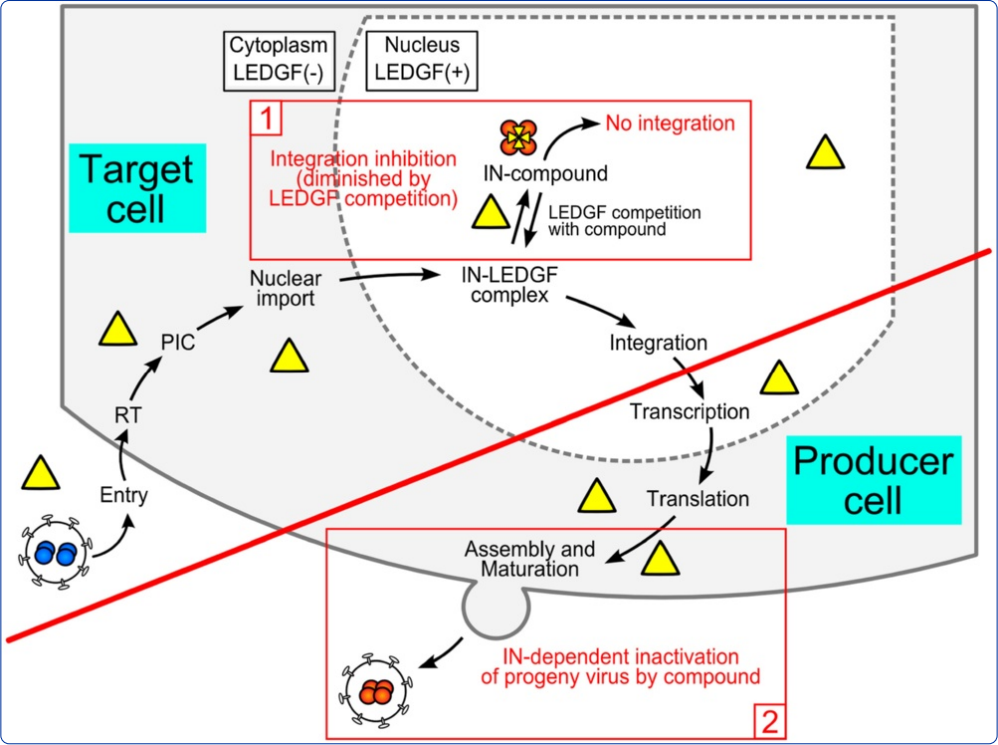
**Model accounting for the discrepancy between the dual ARV activities of INLAIs at integration and post-integration.** The full replication cycle of HIV is represented with steps occurring in target cells **(1)** separated from the steps occurring in virus-producing cells **(2)** by a red line. **(1)** Inhibitory activity at integration and competition by LEDGF in HIV-1 target cells. LEDGF is present as a chromatin-bound protein in the nucleus but is absent in the cytoplasm. After infection of target cells, IN associated with the entering virus (in blue) is imported into the nucleus as part of the pre-integration complex (PIC). Mut101 and INLAIs (yellow triangles) can bind to IN, inhibiting integration by allosteric inhibition of IN strand transfer activity and preventing IN-LEDGF complex formation. In the nucleus of target cells, LEDGF will compete with Mut101 and INLAIs for binding to IN, thus lowering their apparent affinity for IN and counteracting their antiretroviral activity at the integration stage of the replication cycle. **(2)** After integration, progeny virions are assembled in the cytoplasm and at the plasma membrane. INLAIs can bind to the Pol polyprotein precursor containing IN or to the matured IN, in the absence of competing LEDGF. Upon binding, these inhibitors promote conformational modification and enhancement of the IN-IN interaction resulting in IN inactivation (in red). Mut101 and INLAIs activity at the post-integration stage is stronger than their activity at integration as there is no competition with LEDGF in the cytoplasmic cellular compartment.

Mut101 displays weak activity at early stage integration and potent activity at late stage production of defective virions. We then explored how a compound acting on a unique target (IN) and on a unique binding site (the LEDGF-binding pocket), displays such a difference between its potency on two ARV activities. The ARV activity of Mut101 series INLAIs and their inhibition of the IN-LEDGF interaction are clearly linked. There is a tight correlation between their action on IN-LEDGF interaction inhibition and their activity on IN-IN interaction enhancement and IN conformational change. Further studies are required to resolve this issue. However, some clues are provided by Wang *et al*., who studied the ARV activity of a tBPQA compound (racemic BI-D) on wt and LEDGF KO mouse cells infected with a VSV-G-pseudotyped HIV-1 luciferase virus in SR infection experiments [40]. The EC_50_ of racemic BI-D ARV activity was between 2.4 μM and 2.9 μM when tested on wt cells but between 0.16 μM and 0.20 μM (15 to 18 times lower) on LEDGF KO cells, a result not significantly altered by HRP2 disruption. In contrast, the EC_50_ of Raltegravir was similar in each cell type. The authors suggest that LEDGF, present in wt cells but not in LEDGF KO cells, can compete with BI-D for binding to the LEDGF-binding pocket of IN. In the presence of a LEDGF competitor in wt cells, the concentrations of BI-D required to achieve similar ARV activity are higher than when LEDGF is absent in KO cells. Strikingly, we found that the EC_50_ of BI-D ARV activity on MT4 human cells infected with HIV-1 NL4-3 was 2.4 μM ± 0.5 in SR and 0.17 μM ± 0.03 in MR infection assays. This is very similar to the result found by Wang *et al.* (Table 5), although they worked with mouse cells and we worked with human cells. The data strongly suggest that a mechanism similar to that observed by Wang *et al*. (LEDGF competition in SR assay and no competition by LEDGF in MR assay), could explain the difference in ARV activity we found for INLAIs assayed in SR and MR infection assays. These data, and our *in vitro* data showing that LEDGF can compete with Mut101 for binding to IN, support the model illustrated in Figure 8 concerning the considerable difference in the potency of INLAIs between their low ARV activity at integration and their much higher activity inhibiting the production of infectious particles at post-integration stages, although both activities are due to the occupation of the same binding site on IN. The inhibition of HIV-1 integration by INLAIs, measured in SR infection assays, is based on the impairment of the IN-LEDGF interaction and allosteric inhibition of IN. This takes place in the nucleus of HIV-1 target cells. In this cellular compartment LEDGF is abundant and can compete effectively with INLAIs for binding to IN, limiting ARV activity of these inhibitors at this stage. In contrast, the activity of INLAIs at the virus production stage, as measured in MR assays, takes place in the cytoplasm of virus-producer cells after integration. LEDGF, a chromatin-bound nuclear protein, is absent from this cellular compartment and cannot compete with INLAIs for binding to IN or to the Pol polyprotein containing IN [42]. INLAIs are able to target both the IN associated with incoming virions at the step of integration in target cells (in the nucleus, in the presence of competing LEDGF) and the newly synthesized IN in producer cells (associated with progeny virions in the cytoplasm or at the plasma membrane, in the absence of LEDGF). This model suggests that the activity of a protein-protein interaction inhibitor (in this case, concerning the interaction between a viral and a cellular protein) is governed not only by its intrinsic affinity for its target, but also by the cellular compartment in which it is acting. It is the presence or absence of the partner protein of the inhibitor target that could, by competitive binding, negatively affect the level of inhibitor activity.

**Table 5:** **BI-D anti-retroviral activity EC**_**50**_

This study	**Human cells**	
	Single-round/MT4	Multiple-round/MT4
	2.4 ± 0.5 μM	0.17 ± 0.03 μM
Wang *et al.* study [40]	**Mouse cells**	
	LEDGF +/+ WT cells	LEDGF -/- KO cells
	2.4 to 2.9 μM	0.16 to 0.20 μM

A comparison of BI-D ARV activity (EC_50_) assayed in this study (using MT4 human cells) and results reported by Wang *et al*. (using LEDGF WT and KO mouse cells).

The activity of Mut101 and other INLAIs, at the step of integration, may be explained by impairment of IN-LEDGF interaction and their allosteric inhibitory effect on IN strand transfer catalytic activity. However, we need to understand what molecular mode of action of these compounds explains the post-integration block. Gag maturation and CA composition of defective virions produced in the presence of these compounds was normal [42,43] (E. Le Rouzic unpublished results), suggesting that there is no putative effect on maturation of the Gag precursor. We also know that Mut101 does not inhibit viral protease (D. Bonnard unpublished data). A post-integration stage defect could be related to IN conformational change resulting from compound binding to the LEDGF-binding pocket and IN-IN interaction enhancement ([42,43] and this study). We showed, for the first time, that INLAIs promoted long range conformational change when they bind to IN-CCD, affecting residues far away from the compound binding site. Such IN conformational change could negatively affect the formation of the stable synaptic complex (SSC) [37], or influence the currently undefined roles of IN during late stages in the HIV-1 replication cycle [51]. Interestingly, it was lately reported [42-45] that treatment by IN-LEDGF allosteric inhibitors during virus production resulted in a defect in virion morphology with eccentric electron-dense HIV core. Further work is required to answer these questions and defective viruses produced in the presence of Mut101 could be valuable tools for these studies.

The LEDGF-binding pocket lies at the dimeric interface of IN, a region crucial for the formation of an active oligomerization state of IN required for its enzymatic activity and specificity [52-54]. INLAIs make contacts to both subunits of an IN dimer and promote IN conformational change toward inactive oligomers. These inhibitors should therefore be considered as interfacial inhibitors that bind selectively to macromolecular machine interfaces and often promote allosteric effects [55]. Interestingly, INSTIs that bind at the interface of the IN-DNA-Mg^2+^ complex [2] are also considered as archetypal interfacial inhibitors [55].

## Conclusion

The dual mode of action of Mut101 compound series, at two different steps of the HIV replication cycle, is unique and unprecedented in all classes of ARV drugs. This could confer a great advantage to this class of ARV compounds from a therapeutic point of view, provided that clinically efficient concentrations can be reached to inhibit also virus replication at integration. The absence of antagonism between Mut101 compounds and INSTIs or the other classes of drugs currently on the market supports their potential for future ARV therapy.

Several acronyms have been proposed for this class of compounds: LEDGIN [18], NCINI [36] and ALLINI [37] have been suggested to underline their mode of action either as LEDGF-IN inhibitors or as Allosteric IN inhibitors. We would like to propose the acronym of INLAI, standing for ‘IN-LEDGF Allosteric Inhibitor’. This takes into account both the importance of their interference with LEDGF binding to IN and their powerful allosteric inhibitory activity on IN. Our acronym links both activities in the mode of action and highlights that the binding site of these compounds on IN is the LEDGF-binding pocket.

## Methods

### Compound synthesis

Mut029, Mut047, Mut049, Mut062, Mut063, Mut075, and Mut101 compounds were prepared as described in WO2012/140243A1, according to examples 20, 15, 2, 17, 9, 18 and 26, respectively [33]. Details for compound synthesis are given in the Additional file 1. Racemic BI-D was prepared as described in WO2009/062285A1, according to example 41 [20].

### Virology

#### *Reference compounds*

Control compounds such as Saquinavir (SQV), Indinavir (IDV), Nevirapine (NVP), Efavirenz (EFV) and AZT were obtained from the NIH AIDS Research and Reference Reagent Program. Raltegravir (RAL) and Elvitegravir (EVG) were purchased from Selleck Chemicals.

#### *Cell culture*

MT-4, TZM-bl and HeLa-LAV cells were obtained through the AIDS Research and Reference Reagent Program, Division of AIDS, NIAID, NIH. MT-4 cells were grown in RPMI 1640 supplemented with 10% heat-inactivated fetal calf serum and 100 IU/mL penicillin, and 100 μg/mL streptomycin (Invitrogen) to obtain RPMI-complete medium. HeLa-LAV, TZM-bl and 293 T cells (ATCC, CRL-11268) were grown in DMEM supplemented with 10% FCS and antibiotics. TZM-bl cells are a HeLa modified cell line containing separately integrated copies of the luciferase and β-galactosidase genes under control of the HIV-1 promoter.

#### *Virus strains and recombinant HIV-1 molecular clones*

HIV-1 NL4-3 and NL4-3Δ*env*-luc molecular clones were obtained from the NIH AIDS Research and Reference Reagent Program. The SpeI-SalI fragment from pNL4-3 containing the full *pol* gene was cloned into the pUC18 plasmid. *In vitro* mutagenesis was performed with the *Pfu* Turbo (Stratagene) and specific sets of primers to engineer the RT double mutant K103N/Y181C. The mutated fragment was validated by sequencing (Eurofins) and cloned back into pNL4-3 to generate a HIV-1 mutant molecular clone (used as a NNRTI-resistant virus). The molecular clone containing L10R/M46I/L63P/V82T/I84V mutations within the PR-coding region [56] was used as a PR-resistant virus (PI); the clone with M41L/D67N/T69N/K70R/T215F/K219E within the RT-coding region [57] was used as a NRTI-resistant virus; the clone with M41L/D67N/K103N/M184V/L210W/T215Y within the RT-coding region [57] was used as a NRTI and NNRTI-resistant virus (Multi-drug in this study). PI, NRTIs and Multi-drug resistant clones were obtained through the AIDS Research and Reference Reagent Program. The molecular clone containing G140S/Q148H within the IN-coding region obtained from J-F Mouscadet [58] was used as the INSTI-resistant virus.

#### *Viral stock*

293 T (2.2 10^6^ cells) were transfected with 6 μg pNL4–3 proviral plasmids (wild-type or drug resistant) using X-tremeGENE 9 reagent (Roche). Cells were washed 24 h later and cell supernatants were collected 48 h post-transfection and stored at -80°C. Single-round viral stocks were produced by co-transfecting pNL4-3Δ*env* with VSV-G envelope expression vector. Supernatants were collected 2 days after transfection. All viral stocks were quantified for p24 antigen using the Alliance HIV-1 p24 Antigen ELISA (PerkinElmer) and titrated to measure the quantity of infectious particles per mL by infecting TZM-bl indicator cells.

#### *Antiviral assay in MT-4 cells*

MT-4 cells growing exponentially at the density of 10^6^/mL were infected with HIV-1 strain NL4-3 at a MOI (multiplicity of infection) of 0.001 for 2 h. The cells were washed with PBS and aliquoted, using 100 μL fresh complete RPMI, into 96-well white plates (Corning) in the presence of different concentrations of compounds. The effective concentration of compound required to inhibit 50% (EC_50_) of HIV-1 replication was determined after 5 days using the CellTiter-Glo® luminescent reagent (Promega) to quantify cell viability.

#### *Replication-defective-HIV assay*

MT-4 cells (growing exponentially at the density of 10^6^/mL) were infected with VSV-G-pseudotyped NL4-3Δ*env*-luc at a MOI of 0.0001 for 90 minutes. The cells were washed with PBS and aliquoted, using 100 μL fresh complete RPMI, into 96-well white plates (Corning) in the presence of different concentrations of compounds. Luciferase expression was quantified after two days using the One-Glo™ luciferase assay (Promega).

#### *Cytotoxicity assays*

Growth inhibition was monitored in a proliferating human T-cell line (MT-4) with different concentrations of compounds. ATP levels were quantified using the CellTiter-Glo® luminescent reagent (Promega) to measure the ability of a compound to inhibit cell growth, an indication of the compound’s cytotoxicity. Cytotoxicity was evaluated at either day 2 or day 5.

#### *Time-of-addition experiment*

MT-4 cells in a 96-well microtiter plate (10^5^ cells per well) were infected with pseudotyped HIV-1 NL4-3 strain at a MOI of 0.001. Compounds were added to single-round infection assays at different time points after infection (0, 2, 3, 4, 5, 6, 8 and 24 h). RAL, NVP and Mut101 were added at 80 nM, 2 μM and 25 μM, respectively. This corresponded to between three and ten times their EC_50_ as determined by a drug susceptibility assay (CT-Glo).

#### *Quantification of viral cDNA by real-time PCR*

Prior to infection, viral stocks were treated 1 h at 37°C with 100 U per mL of DNAseI (Roche Applied Science). MT4 cells (6x10^6^) were infected with virus at MOI = 0.001. At 7 h, 24 h and 48 h post-infection, cells were harvested, washed twice in PBS and DNA was extracted using the QIAamp Blood DNA Minikit (Qiagen). Quantifications of viral DNA were performed by real-time PCR using the LightCycler 480 system (Roche Applied Science). Primers, probes, and PCR run conditions were described previously [59]. The copy number of HIV-1 late reverse transcription product (LRT) was determined using standard curves obtained by amplification of cloned DNA containing the matched sequences. The copy number of integrated HIV-1 DNA was determined in reference to a standard curve generated by concomitant two-stage PCR amplification of a serial dilution of the standard HeLa HIVR7-Neo cell DNA [59]. Copy numbers of each viral form were normalized with the number of cells obtained by the quantification by PCR of the β-globin gene according to the manufacturer instructions (Roche Applied Science).

### Molecular biology and biochemistry

#### *Constructions of epitope-tagged proteins*

The His_6_-LEDGF plasmid has been previously described [60]. The plasmid encoding GST-Flag-IBD/LEDGF was constructed by cloning the LEDGF DNA sequence (encoding residues 342 to 507) in fusion with the Flag epitope into pGEX-2 T (GE Healthcare). His_6_-IN plasmid corresponds to pINSD.His and has been previously described [61]. The IN A128T mutant was generated by site-directed mutagenesis from pINSD.His. The full length Flag-tagged integrase sequence from NL4-3 was PCR amplified and cloned between the BamHI and XhoI restriction sites of a pGEX-6P1 vector (GE Healthcare) to generate the expression plasmid GST-Flag-IN. His-CCD and GST-Flag-CCD were obtained by cloning the integrase region (residues 50 to 202, encoding the catalytic core domain) from pINSD.His.Sol [62] into pET15b and pGEX-2 T-Flag, respectively. CCD contains the F185K mutation which greatly improves the solubility of the recombinant protein. The CCD T174I mutation was introduced into the His-CCD plasmid by site-directed mutagenesis.

#### *Purification of recombinant proteins*

Frozen cells pellets from one liter culture were resuspended in 3.5 mL of integrase buffer (50 mM HEPES pH 7.5, 1 M NaCl, 7 mM CHAPS, 5 mM MgCl_2_, 2 mM β-mercaptoethanol, 10% glycerol) (for full length integrase) or the same buffer in a two-fold water dilution (for integrase CCD), containing Complete™ protease inhibitor cocktail (Roche) and benzonase (Sigma). Cells were disrupted using 25 g - 30 g, 150–212 μm glass beads (Sigma) and vortexed at 4°C for 10 min. Glass beads were washed three times with 15 mL extraction buffer and whole cell lysate was centrifuged at 109,000 g (R_max_) for 1 h at 4°C in a Beckman XL80K ultracentrifuge.

His_6_-tagged IN wt or A128T, or His_6_-tagged IN-CCD lysate was loaded at 3 mL/min on a 5 mL His-Trap FF crude column (GE Healthcare) previously equilibrated with integrase buffer or CCD buffer, respectively, containing 20 mM imidazole. Samples were washed until OD_280nm_ returned to baseline and bound proteins were then eluted using a 20 to 500 mM imidazole gradient over 20 column volumes. Pooled fractions were concentrated to 2.5 mL using Amicon Ultra 15™ 10 K centrifugal filter devices (Millipore) at 4,000 g and 4°C. Concentrated protein was loaded on a Superdex 200 16/600 PG column (for full length IN) or a Superdex 75 16/600 PG column (for IN-CCD) (GE Healthcare), previously equilibrated with integrase buffer at 4°C. Chromatography was performed at 4°C. The presence of His_6_-Tag IN/CCD in collected fractions was assessed by electrophoresis on NuPAGE Bis-Tris 10% acrylamide gels with MES as the electrophoresis buffer (Invitrogen). Proteins were stained using Imperial Protein Stain^TM^ (Thermo Scientific Pierce). Pooled fractions from Superdex200 or Superdex75 separation were concentrated and stored at -80°C until further use. GST-tagged Flag-CCD and GST-tagged Flag-IBD lysates were loaded at 0.25 mL/min on a 20 mL Glutathione Sepharose 4 Fast Flow (GE Healthcare) column. Bound proteins were eluted using integrase CCD buffer with 20 mM reduced glutathione. Purification was completed as described above. Flag-IN was prepared from a GST-Flag-IN fusion protein using the pGEX-6P expression system (GE Healthcare). After adsorption to the Glutathione Sepharose 4 Fast Flow column, protein corresponding to the 1 liter culture extract was digested by 250 units of PreScission Protease (GE Healthcare) for 16 hours at 4°C. Cleaved protein was eluted by restarting the buffer flow over the column. Purification was carried out by gel filtration on Superdex 200, as described above. rGST was purified on Glutathione Sepharose 4 Fast Flow and Superdex 75 16/600 PG columns as described above but using a PBS buffer.

#### *HTRF®-based CCD-IBD interaction assay*

All HTRF® conjugated monoclonal antibodies were purchased from Cisbio Bioassays. IN-CCD/LEDGF-IBD HTRF® assay was performed in 384-well low volume black polystyrene plates (Corning) in CCD-IBD assay buffer (25 mM HEPES pH 7.4, 150 mM NaCl, 2 mM MgCl_2_, 0.4 M KF, 0.1% bovine serum albumin, 1 mM DTT). 2 μL of 3-fold serial dilutions of inhibitory compound in 25% DMSO were preincubated for 30 min at room temperature with 8 μL of IN-CCD mixture (75 nM His_6_-IN-CCD, 17 nM XL_665_-conjugated anti-His_6_ monoclonal antibody). Then, 10 μL of LEDGF-IBD mixture (20 nM GST-Flag-LEDGF-IBD, 1.8 nM Europium cryptate-labelled anti-GST monoclonal antibody) were added and the plate was incubated for 2.5 h at room temperature before reading the time-resolved fluorescence in a PHERAstar Plus (BMG Labtech) with HTRF module (excitation at 337 nm, dual emission at 620 nm and 667 nm). The HTRF ratio was converted to % inhibition and analyzed by fitting with a sigmoidal dose–response equation with Hill slope to determine the compound IC_50_.

#### *HTRF®-based IN-LEDGF interaction assay*

IN-LEDGF HTRF® assay was performed in 384-well low volume black polystyrene plates (Corning) using IN-LEDGF assay buffer (25 mM Tris–HCl pH 7.4, 150 mM NaCl, 2 mM MgCl_2_, 0.4 M KF, 0.1% Igepal CA-630, 0.1% bovine serum albumin, 1 mM DTT). 2 μL of 3-fold serial dilutions of inhibitory compound in 25% DMSO were preincubated for 30 min at room temperature with 8 μL of IN mixture (50 nM Flag-tagged IN, 17 nM XL_665_-conjugated anti-Flag M2 monoclonal antibody). 10 μL of LEDGF mixture (60 nM His_6_-tagged LEDGF/p75, 1.5 nM Terbium cryptate-labelled anti-His_6_ monoclonal) were added and the plate was incubated for 2.5 h at room temperature before reading the time-resolved fluorescence in a PHERAstar Plus with HTRF module (excitation at 337 nm, dual emission at 620 nm and 667 nm). The HTRF ratio was converted to % inhibition and analyzed by fitting a sigmoidal dose–response equation with Hill slope to determine the IC_50_ of the compound.

For the LEDGF competition assay, an IN-LEDGF assay was performed with various concentrations of His_6_-LEDGF in the LEDGF mixture (from 15 nM to 0.96 μM).

#### *HTRF®-based IN multimerization assay*

IN-IN HTRF® assay was performed in 384-well low volume black polystyrene plates (Corning). 2 μL of 3-fold serial dilutions of inhibitory compound in 25% DMSO were preincubated for 30 min at room temperature with 4 μL of 125 nM Flag-IN dilution. 4 μL of 125 nM His_6_-IN were added and the plate was incubated for 3 h at room temperature to allow IN subunit exchange and multimerization. This step was performed in IN2 buffer (25 mM HEPES pH 7.4, 150 mM NaCl, 2 mM MgCl_2_, 0.005% Tween-20, 0.1% bovine serum albumin, 1 mM DTT). 10 μL of revelation mixture (1.1 nM Europium cryptate-labelled monoclonal anti-Flag M2 antibody and 13 nM XL_665_-labeled anti-His_6_ monoclonal antibody in IN2 buffer supplemented with 0.8 M KF) were added and the plate was incubated for 2 h at room temperature before reading the time-resolved fluorescence in a PHERAstar Plus with HTRF module (excitation at 337 nm, dual emission at 620 nm and 667 nm). The HTRF ratio was converted to % activation and analyzed by fitting a sigmoidal dose–response equation with Hill slope to determine the AC_50_ of the compound and the activation plateau.

#### *IN strand transfer ELISA assay*

IN strand transfer ELISA assay has been adapted from [63]. The strand transfer reaction was performed in 96-well V bottom polypropylene microplates (Greiner Bio-One) containing 4 μL of 3-fold serial dilutions of compound or 25% DMSO. 16 μL of IN mixture (20 mM HEPES pH 7.5, 10 mM MgCl_2_, 1 mM DTT, 0.50 μM His_6_-IN) was added. After a 15 min preincubation, 20 μL of substrate oligonucleotide mixture (0.20 μM Biotin-LTR preprocessed donor DNA, 0.20 μM Digoxigenin (DIG)-Target DNA) was added and the plate was incubated for 2 h at 37°C. The reaction was stopped by addition of 60 μL stop mixture (20 mM Tris–HCl pH 7.6, 0.4 M NaCl, 10 mM Na_2_EDTA, 0.1 mg/mL salmon sperm DNA) and the volume transferred to Reacti-Bind high-binding capacity streptavidin-coated white plates (Thermo Scientific Pierce). After 1 h incubation at room temperature under gentle shaking, integrase and unjoined DNA were removed by three washes with 200 μL wash solution 1 (30 mM NaOH, 0.2 M NaCl, 1 mM Na_2_EDTA). 100 μL of 2000-fold diluted HRP-conjugated anti-DIG Fab (Roche Applied Science) was added and the plate was incubated for 1 h at 37°C. Unbound antibody was removed with wash solution 2 (PBS pH 7.4, 0.05% Tween-20, 0.1% bovine serum albumin), 100 μL of SuperSignal Femto ELISA substrate (Thermo Scientific Pierce) was added and chemiluminescence was immediately read in a PHERAstar Plus with LUM-plus module. The signal, converted to % inhibition, was analyzed by fitting a sigmoidal dose–response curve to determine IC_50_ and the inhibition plateau.

#### *IN 3′ processing and strand transfer radioactive assays*

Sequences of the different oligonucleotides (ODN) substrates are U5B: 5′-GTGTGGAAAATCTCTAGCAGT-3′, U5A: 5′- ACTGCTAGAGATTTTCCACAC-3′ and U5B-2: 5′-GTGTGGAAAATCTCTAGCA-3′. ODNs were purchased from Eurogentec and further purified by electrophoresis in a denaturing 16% acrylamide/urea gel. For activity assays, ODNs (U5B and U5B-2) were radiolabelled with T4 polynucleotide kinase (New England Biolabs) and γ[^32-P^]ATP (3000 Ci/mmol) (Amersham), and purified on a Sephadex G-10 column (GE Healthcare). Double-stranded ODNs (U5B with U5A and U5B-2 with U5A used for 3′-processing and strand transfer reactions, respectively) were obtained by mixing equimolar amounts of complementary strands in the presence of 100 mM NaCl.

IN activity assays – 3′-processing, strand transfer – were carried out at 37°C with the full-length HIV-1 IN, in a buffer containing 10 mM HEPES (pH 7.2), 1 mM DTT, 7.5 mM MgCl_2_ in the presence of 6.25 nM DNA (3′-processing) or 12.5 nM DNA (strand transfer) as described previously [64]. For negative control, 100 mM Na_2_EDTA was added to the reaction before incubation. Products were separated by electrophoresis in denaturing 16% acrylamide/urea gels. Gels were analysed with a Molecular Dynamics STORM phosphoimager and quantified with ImageQuant™ 4.1 software.

#### *Size exclusion chromatography (SEC) experiments with IN liganded with Mut101 and BI-D compounds*

SEC was performed with a Superdex 200 10/300 GL column (GE Healthcare) using a flow-rate of 0.4 mL/min in buffer containing 50 mM HEPES, pH 7.5, 1 M NaCl, 7 mM CHAPS, 5 mM MgCl_2_, 10 mM DTT, 10% glycerol at room temperature. His_6_-IN wt (21 μM) or His_6_-IN A128T (40 μM) was incubated for 10 min with 100 μM BI-D or Mut101 before injection on the column. Protein elution was monitored at 280 nm.

#### *Biacore experiments*

Experiments were carried out using a Biacore 3000 instrument (GE Healthcare) at 25°C. An anti-GST antibody (GST Capture Kit, GE Healthcare) was immobilized on two flow-cells of a CM5 sensor chip by amine coupling according to the recommendations of the manufacturer. GST-Flag tagged IN CCD proteins (wild type and T174I mutant) at 68 μg/mL in HBS-EP buffer (GE Healthcare) were captured on one flow-cell (8 min injection at 10 μL/min) while recombinant GST (60 μg/mL in HBS-EP buffer, 8 min injection at 10 μL/min) was injected on the other flow-cell and used as a reference. Kinetics experiments with Mut101 were carried out at 60 μL/min with a 3 min injection of each dilution of the compound in HBS-EP followed by 10 min dissociation. Sensorgrams were evaluated using BiaEvaluation 3.2 software.

### Structural studies

Crystallization was performed by the hanging-drop vapor-diffusion method at 297 K in 24-well plates. The catalytic domain (CCD) of HIV-1 IN with mutation F185K was expressed and purified as previously described [62]. Prior to any crystallization experiment, the protein was simultaneously dialyzed and concentrated at 277 K with an Amicon Ultra-10 device (Millipore) equipped with a 10 kDa cut-off dialysis membrane. The dialysis solution was 50 mM MES-NaOH pH 5.5, 50 mM NaCl and 5 mM DTT. The protein was concentrated to between 3 mg/mL and 5 mg/mL.

Each hanging-drop consisted of 3 μL protein solution and 3 μL reservoir solution, with 500 μL reservoir solution in the well. Initial screening was carried out using Qiagen kits (Classics & JCSG+) and positive hits were then optimized. The optimized reservoir solution consisted of 1.16-1.36 M ammonium sulfate, 50 mM sodium cacodylate-HCl pH 6.5. The crystals grew to approximate dimensions of 0.2 x 0.2 x 0.4 mm within one week. They were soaked with the Mut101 ligand for 5 days before data collection by adding a 10 mM stock solution of the inhibitor to the drop. The crystals were plunged in oil (FOMBLIN Y LVAC 14/6 from Aldrich) for a few seconds and cryo-cooled in a stream of liquid nitrogen at 100 K. All data were collected at a temperature of 100 K and processed with XDS [65]. All diffraction data were collected using a Pilatus 2 M detector on beamline X06DA (PXIII) at the Swiss Light Source, Paul Scherrer Institut, Villigen, Switzerland. Structure determination was carried out using the CCP4 suite of programs [66]. The structures of the integrase, both in complex with the Mut101 inhibitor or not, were determined by molecular replacement using the program MOLREP [67] and PDB entry 1BHL [68] as the starting model. The models were built manually using the program Coot [69] and refined with the program REFMAC [70]. Arp/Warp [71] was used for the automatic ligand [72] and water molecule fitting. Structures and structure factors have been deposited in the PDB with codes 4LH4 (IN CCD) and 4LH5 (IN CCD with Mut101 inhibitor).

All experiments have been performed under Authorization Number 5606 CA-I, assigned by the French Ministry of Research for work with genetically modified organisms.

## Competing interests

ELR, DB, SC, JMB, FC, FLS, JN, RBea, CA, JB, SV, BL, FM, and RBen declare that they are full time employees of Biodim-Mutabilis. SEil, MR, NL, OD, ED, AS, AZ, and SEmi declare that they have no competing interests.

## Authors’ contribution

ELR, JN and RBea generated plasmid constructs, viruses and virus infected cells and conducted all virology tests. ELR, SEmi, AS, AZ, FM and RBen designed the studies of antiretrovirals and the selection of resistant mutant viruses. SEmi and CA designed and performed the qPCR studies. ELR designed figures and helped to draft the manuscript. DB designed and performed HTRF and *in vitro* activity studies, designed figures and helped to draft the manuscript. OD designed and performed the radioactive IN activity studies. JMB designed and performed the preparation and purification of recombinant proteins, size exclusion chromatography and Biacore SPR studies, and helped to draft the manuscript. SC designed and conducted compound synthesis and studies with BL, FC, FLS, JB and SV. MR designed the co-crystallization and structural studies and performed these with SEil. NL, FM and RBen designed and coordinated the full study and drafted the manuscript. All authors read and approved the final manuscript.

## Supplementary Material

Additional file 1 Additional methods, figures and tables. Click here for file

Additional file 2 **The movie was generated with PyMOL **[73]**.** The intermediate structures between the initial and final state where generated using the morphing option in Pymol. The two IN monomers are colored in red and gold. The magnesium ion is represented as a green sphere and the coordinating residues of the magnesium and in the Mut101 pocket are represented in sticks. The solvent accessible surface coloring is in red and gold for the carbon atom of the corresponding monomer with the nitrogen in blue, the oxygen in red and sulfur in yellow. Mut101 is represented in cyan. Click here for file
