# Supplementary material for: Dual inhibition of HIV-1 replication by integrase-LEDGF allosteric inhibitors is predominant at the post-integration stage
Source: Retrovirology. 2013 Nov 21;10:144. doi: 10.1186/1742-4690-10-144 (PMC4222603; doi:10.1186/1742-4690-10-144)
Supplement: Additional file 1 — Additional methods, figures and tables. [file 1742-4690-10-144-S1.doc]

**Additional file S1: Supplemental Information**

**Supplementary methods:**

**Compound synthesis:**

**Synthesis of Mut029 (2-(*tert*-butoxy)-2-[6-(3,4-dihydro-2*H*-1-benzopyran-6-yl)quinolin-5-yl]acetic acid)**

Mut029 compound is prepared as shown in scheme 1 and the full experimental details are available as example 20 in patent WO2012/140243A1.

**Scheme 1**

**Synthesis of Mut047 (2-(*tert*-butoxy)-2-[2-(3,4-dihydro-2H-1-benzopyran-6-yl) naphthalene-1-yl]acetic acid)**

Mut047 compound is prepared as shown in scheme 2 and the full experimental details are available as example 15 in patent WO2012/140243A1.

**Scheme 2**

**Synthesis of Mut049 (2-(*tert*-butoxy)-2-(4-cyclohexyl-2-methylquinolin-3-yl)acetic acid)**

Mut049 compound is prepared as shown in scheme 3 and the full experimental details are available as example 2 in patent WO2012/140243A1.

**Scheme 3**

**Synthesis of Mut062 (2-(*tert*-butoxy)-2-[4-(3,4-dihydro-2*H*-1-benzopyran-6-yl)-2*H*-chromen-3-yl]acetic acid)**

Mut062 compound is prepared as shown in scheme 4 and the full experimental details are available as example 17 in patent WO2012/140243A1.

**Scheme 4**

**Synthesis of Mut063 (2-(*tert*-butoxy)-2-[2-(3,4-dihydro-2*H*-1-benzopyran-6-yl)-4-phenoxyphenyl]acetic acid)**

Mut063 compound is prepared as shown in scheme 5 and the full experimental details are available as example 9 in patent WO2012/140243A1.

**Scheme 5**

**Synthesis of Mut075 (2-(*tert*-butoxy)-2-(2-methyl-4-phenyl-5,6,7,8-tetrahydro-quinolin-3-yl)acetic acid)**

Mut075 compound is prepared as shown in scheme 6 and the full experimental details are available as example 18 in patent WO2012/140243A1.

**Scheme 6**

**Synthesis of Mut101 (2-(*tert*-butoxy)-2-[4-(3,4-dihydro-2*H*-1-benzopyran-6-yl)-2-methyl-5,6,7,8-tetrahydroquinolin-3-yl]acetic acid)**

Mut101 compound is prepared as shown in scheme 7 and the full experimental details are available as example 26 in patent WO2012/140243A1.

**Scheme 7**

**Supplementary tables:**

**Table S1. Data collection and refinement statistics.** Each data set was collected from one single crystal. Values in parentheses are for the outer shell.

|  | IN-CCD (50-212)  (PDB entry 4LH4) | Mut101/IN-CCD (50-212)  (PDB entry 4LH5) |
| --- | --- | --- |
| Data collection |  |  |
| Xray wavelength (Å) | 0.9191 | 1.0000 |
| Space group | *P3_1_21* | *P3_1_21* |
| Unit cell parameters (Å) | a=b=72.53 ; c=64.92  α=β=90 ; γ=120 | a=b=72.94 ; c=65.88  α=β=90 ; γ=120 |
| Resolution range (Å) | 50–1.85 (1.96–1.85) | 50–2.19 (2.33–2.19) |
| No. of measured reflections | 162,265 (17,941) | 66,092 (9,901) |
| No. of unique reflections | 29,173 (3,469) | 19,590 (2,971) |
| Completeness (%) | 88.7 (65.0) | 97.6 (93.4) |
| Mean I/σ (I) | 24.14 (6.76) | 25.20 (8.96) |
| Multiplicity | 5.56 (5.17) | 3.37 (3.33) |
| R*_merge_* (%) | 3.9 (14.1) | 2.9 (12.3) |
| Refinement statistics |  |  |
| Resolution range(Å) | 28.27–1.80 (1.85–1.80) | 45.56–2.19 (2.25–2.19) |
| Reflections used | 16,757 (1,295) | 10,071 (665) |
| R factor (%) | 20.222 (25.900) | 18.067 (25.3) |
| R*_free_* (%) | 24.625 (34.000) | 23.625 (35.4) |
| No. of protein atoms | 1,190 | 1,152 |
| No. of ligand atoms | 0 | 30 |
| No. of ions | 1 | 1 |
| No. of water | 169 | 91 |
| R.m.s. deviation |  |  |
| Bond lengths (Å) | 0.023 | 0.020 |
| Bond angles (°) | 2.354 | 2.614 |
| Ramachandran plot (%) |  |  |
| Most favoured region | 97.5 | 97.5 |
| Additionally allowed region | 2.5 | 2.5 |

**Table S2. Size exclusion chromatography results.** Elution volume of the different peaks shown on Figure 4C-F with the corresponding MWs according to the elution volumes of the protein markers used, and the extrapolated putative oligomeric states of IN. Elution volumes for molecular weight standards during column calibration were aldolase (158 kDa) 12.17 mL; conalbumin (75 kDa) 13.7 mL; carbonic anhydrase (29 kDa) 16.23 mL; and ribonuclease A (13.7 kDa) 17.62 mL.

| Peak no. | Elution volume  (mL) | Estimated MW  (kDa) | Estimated oligomeric state |
| --- | --- | --- | --- |
| 1 | 14.65 | 52.6 | Dimer |
| 2 | 12.86 | 114.7 | Mix of dimer and higher oligomer |
| 3 | 13.0 | 107.9 | Mix of dimer and higher oligomer |
| 4 | 14.80 | 49.2 | Dimer |
| 5 | 12.51 | 133.5 | Tetramer |
| 6 | 12.68 | 124 | Tetramer |
| 7 | 7.5 | ≥1000 | Soluble aggregates |

**Supplementary figures:**

**Figure S1**

**Figure S1. (A) Overall structure of the IN-CCD dimer in complex with Mut101.** One monomer is in gold, the other in blue. **(B)** Electrostatic potential map. The negative potential is in red, the positive in blue and the neutral in grey. The ligand is shown in green sticks with the oxygen in red and the nitrogen in blue**. (C)** 2D view showing the interaction (hydrophobic and H-bonds between the ligand, protein and two water molecules).The figure was made using with PyMOL (DeLano, 2002). The electrostatic potential has been calculated using the Adaptive Poisson-Boltzmann Solver (APBS) software (Baker et al., 2001) imbedded in PyMOL and the 2D view of the interactions was generated using LigPlot+ (Laskowski and Swindells, 2011).

**Figure S2**

**Figure S2. Structure of racemic BI-D.**

**Supplementary references:**

Baker, N.A., Sept, D., Joseph, S., Holst, M.J., and McCammon, J.A. (2001). Electrostatics of nanosystems: application to microtubules and the ribosome. Proc Natl Acad Sci U S A *98*, 10037-10041.

DeLano, W.L. (2002). The PyMOL Molecular Graphics System (Delano Scientific, Paolo Alto, CA).

Laskowski, R.A., and Swindells, M.B. (2011). LigPlot+: multiple ligand-protein interaction diagrams for drug discovery. J Chem Inf Model *51*, 2778-2786.
